# IFN-γ and IL-21 Double Producing T Cells Are Bcl6-Independent and Survive into the Memory Phase in *Plasmodium chabaudi* Infection

**DOI:** 10.1371/journal.pone.0144654

**Published:** 2015-12-08

**Authors:** Victor H. Carpio, Michael M. Opata, Marelle E. Montañez, Pinaki P. Banerjee, Alexander L. Dent, Robin Stephens

**Affiliations:** 1 Department of Internal Medicine, Division of Infectious Diseases, University of Texas Medical Branch, Galveston, Texas, United States of America; 2 Department of Microbiology and Immunology, University of Texas Medical Branch, Galveston, Texas, United States of America; 3 Center for Human Immunobiology of Texas Children’s Hospital, Baylor College of Medicine, Houston, Texas, United States of America; 4 Department of Microbiology and Immunology, Indiana University School of Medicine, Indianapolis, Indiana, United States of America; Aaron Diamond AIDS Research Center with the Rockefeller University, UNITED STATES

## Abstract

CD4 T cells are required to fight malaria infection by promoting both phagocytic activity and B cell responses for parasite clearance. In *Plasmodium chabaudi* infection, one specific CD4 T cell subset generates anti-parasitic IFN-γ and the antibody-promoting cytokine, IL-21. To determine the lineage of these multifunctional T cells, we followed IFN-γ^+^ effector T cells (Teff) into the memory phase using *Ifng*-reporter mice. While *Ifng* ^+^ Teff expanded, the level of the Th1 lineage-determining transcription factor T-bet only peaked briefly. *Ifng* ^+^ Teff also co-express ICOS, the B cell area homing molecule CXCR5, and other Tfh lineage-associated molecules including Bcl6, the transcription factor required for germinal center (GC) T follicular helper cells (Tfh) differentiation. Because Bcl6 and T-bet co-localize to the nucleus of *Ifng* ^+^ Teff, we hypothesized that Bcl6 controls the Tfh-like phenotype of *Ifng* ^+^ Teff cells in *P*. *chabaudi* infection. We first transferred Bcl6-deficient T cells into wildtype hosts. Bcl6-deficient T cells did not develop into GC Tfh, but they still generated CXCR5^+^IFN-γ^+^IL-21^+^IL-10^+^ Teff, suggesting that this predominant population is not of the Tfh-lineage. IL-10 deficient mice, which have increased IFN-γ and T-bet expression, demonstrated expansion of both IFN-γ^+^IL-21^+^CXCR5^+^ cells and IFN-γ^+^ GC Tfh cells, suggesting a Th1 lineage for the former. In the memory phase, all *Ifng* ^+^ T cells produced IL-21, but only a small percentage of highly proliferative *Ifng* ^+^ T cells maintained a T-bet^hi^ phenotype. In chronic malaria infection, serum IFN-γ correlates with increased protection, and our observation suggests *Ifng* ^+^ T cells are maintained by cellular division. In summary, we found that *Ifng* ^+^ T cells are not strictly Tfh derived during malaria infection. T cells provide the host with a survival advantage when facing this well-equipped pathogen, therefore, understanding the lineage of pivotal T cell players will aid in the rational design of an effective malaria vaccine.

## Introduction

Immunity against intracellular pathogens, such as the blood-stages of the rodent malaria parasite *Plasmodium chabaudi*, requires both antibodies and Th1-type responses [1, 2]. This rodent parasite shares many traits with human malaria parasites, such as *P*. *falciparum*, and generates immunity that parallels that observed in human malaria [3]. The CD4 T cell response starts with strong IFN-γ production, which reduces the initial parasite growth, followed by a marked change in the response to promote antibody and B cell involvement. This is critical because antibody is required for complete parasite clearance [4]. CD4 T cells isolated from *P*. *chabaudi* infected mice on day 40 and cultured for two weeks with parasite antigen lose their IFN-γ production capacity, but gain the ability to generate IL-4 and provide help to B cells, suggesting that they are not of pure Th1 lineage [4].

The transition from Th1 to antibody promoting T cells in response to *P*. *chabaudi* is likely regulated by B cells, as T cells from infected B cell deficient (muMT) mice produce more IFN-γ and less IL-4, and become inefficient to help antibody formation [5]. Furthermore, during the early phases of this infection there is a switch in the type of antigen presenting cells, which reduces IFN-γ production [6]. This change in T cell function includes acquiring the ability to secrete the regulatory cytokine IL-10, and the antibody-promoting cytokine IL-21 [7, 8]. This response seems appropriate to achieve an adequate balance between parasite control and immunopathology. Despite this controlled regulation, serum IFN-γ and IFN-γ^+^ T cells correlates with resistance to *P*. *falciparum* in African children [9, 10]. Therefore, understanding the generation of IFN-γ-producing memory T cells is important for the rational creation of a malaria vaccine.

It was recently reported that IL-21 generated by IFN-γ^+^IL-10^+^ T cells is critical to generate antibodies that control chronic infection and re-infection [8]. This new data suggests that the earlier reported switch from IFN-γ^+^ Th1 immunity relates to an increase in CXCR5^+^IL-21^+^ T follicular helper cells (Tfh) [11]. Indeed, a recent study in Malian children uncovered that CXCR5^+^PD-1^+^CXCR3^+^ Th1-like Tfh cells are the predominant response against acute malaria. Importantly, these Th1-like Tfh cells were unable to mount an optimal antibody response, albeit produced the highest levels of IL-21 [12]. Th1 cells are the major source of IL-10 during this infection, as in other chronic parasitic infections, and it is induced by IL-27 [7, 13–15]. Importantly, IL-27 can also induce IL-21 [16], and promote Tfh development [17]. The transcriptional regulation of IL-21 expression in T cells is not clearly defined and may involve Bcl6, as well as Maf and STAT3 [18–20].

IL-21 has a pivotal role in B cell differentiation and germinal center formation, but can also have effects on T cell biology, including inhibition of IFN-γ production [21]. However, this finding may be limited in scope as CD4 T cells cultured *in vitro* under Th1 polarizing conditions can produce significant levels of IL-21 [18]. Conversely, although IL-21 is the signature cytokine of the Tfh subset [22], these cells can simultaneously express other cytokines, including IFN-γ, depending on the nature of the cytokine milieu [23]. For example, experiments using an influenza infection model in IL-21 reporter mice showed that CXCR5^+^PD-1^+^IL-21^+^ Tfh cells can express IFN-γ, IL-10, and T-bet [24]. Therefore, it is not clear whether the unusually large amount of IL-21 observed in this chronic infection is made by Tfh- or Th1-lineage derived cells, and if they are able to survive into the memory phase.

Herein, we investigated IFN-γ-producing effector T cells elicited during *P*. *chabaudi* infection for molecular evidence of Th1 commitment, and their ability to generate IFN-γ^+^ IL-21^+^memory T cells. Using an *Ifng*/*Thy1*.*1* reporter mouse, we observed that a majority of IFN-γ^+^ T cell responders expressed several Tfh markers. In line with previous findings [8, 12], the dominant IFN-γ^+^ Teff population identified was CXCR5^+^, and these cells produced high levels of IFN-γ, in addition to IL-10 and IL-21. An IFN-γ^+^ CXCR5^hi^PD-1^hi^ IL-21^+^ GC Tfh population was also observed. The CD4^+^IFN-γ^+^ effector T cells also expressed both T-bet and the Tfh lineage-promoting transcription factor Bcl6. As expected, deficiency of Bcl6 regulated the CXCR5^hi^PD-1^hi^ GC Tfh subset. On the other hand, Bcl6 did not regulate the CXCR5^+^IL-21^+^IFN-γ^+^ population. We also studied IL-10 deficient mice, which have increased T-bet and IFN-γ in T cells to promote Th1 development. We found that in response to *P*. *chabaudi* infection, these mice generated increased levels of both CXCR5^+^IL-21^+^IFN-γ^+^ T cells and IFN-γ^+^ GC-Tfh. During the memory phase, we found that IFN-γ^+^ T cells at day 60 post-infection were able to produce IL-21. Adoptive transfer of CFSE-labeled IFN-γ^+^ T cells revealed that T-bet and IFN-γ expression are only maintained by cell division in the memory phase. Together, these findings suggest that a heterologous T helper memory cell population is critical to the malaria immune response because it maintains both cellular and humoral immunity through IFN-γ, IL-21, and CXCR5, and regulates pathology via IL-10. Importantly, this subset is not dependent on Bcl6 suggesting is not of the Tfh lineage. These results have significant implications for our understanding of the protective responses against malaria, and intend support the development of effective vaccines to control and prevent malaria.

## Materials and Methods

### Animals and infections

C57BL/6J (B6), B6.SJL-*Ptprc* ^*a*^ *Pepc* ^*b*^/BoyJ (CD45.1) and B6.129P2-*Il10* ^*tm1Cgn*^/J (IL-10 deficient) were purchased from The Jackson Laboratory (Bar Harbor, ME), and CD4-Cre^+^ mice from Taconic (Hudson, NY). *Ifng/Thy1*.*1* Knock-In and *Ifng/Thy1*.*1* BAC-In mice were a kind gift of Casey Weaver (University of Alabama, Birmingham, AL). Bcl6^fl/fl^ x CD4-Cre mice [25] (Indiana University School of Medicine, Indianapolis, IN) were bred at UTMB. The floxed allele was genotype by PCR using the primers for the 3’ loxP site: forward 5’-TCACCA ATCCCAGGTCTCAGTGTG-3’; reverse 5’-CTTTGTCATATTTCTCTGGTTGCT-3’. All mice were maintained in our specific pathogen free animal facility with *ad libitum* access to food and water. Mice 6–12 weeks old were infected with 10^5^ *Plasmodium chabaudi chabaudi* (AS) courtesy of Jean Langhorne (MRC NIMR, London, UK) infected erythrocytes i.p. Parasites were counted in thin blood smears stained with Giemsa (Sigma, St. Louis, MO) by light microscopy [26].

### Animal Care Statement

All animal experiments were carried out in compliance with the protocol specifically approved for this study by the University of Texas Medical Branch Institutional Animal Care and Use Committee.

### Flow Cytometry and Imaging Flow Cytometry

Single-cell suspensions from spleens were made in HEPES buffered Hank’s Balanced Salt Solution (Gibco, Lifetechnologies, Grand Island, NY), incubated in red blood cell lysis buffer (eBioscience, San Diego, CA), and stained in PBS 2% FBS (Sigma, St. Louis, MO) and 0.01% sodium azide with anti-CD16/32 (2.4G2) supernatant (BioXcell, West Lebanon, NH) followed by combinations of FITC–, PE–, PerCP-Cy5.5, PE/ Cyanine 7 (Cy7), Allophycocyanin monoclonal antibodies (all from eBioscience, San Diego, CA), CD127-PE/Cy5, CD44-Brilliant Violet 785 (BV785), CXCR3 BV421 (Biolegend, San Diego, CA), CXCR5-Biotin (BDbioscience, San Jose, CA) followed by either Streptavidin-eFluor 450,–PE or–BV650. For experiments using KI and Bcl6^fl/fl^CD4^Cre^ mice, CXCR5 staining was performed using rat anti-mouse purified CXCR5 (BDbioscience, San Jose, CA) for 1 hour at 4°C followed by 30 min incubation with biotin conjugated AffiniPure Goat anti-rat (H+L) (Jackson Immunoresearch, West Grove, PA) followed by Streptavidin [27]. For intracellular staining, total cells were stimulated for 2 h with PMA (50 ng/mL), ionomycin (500 ng/mL), and Brefeldin A (10 μg/mL) in complete Iscove’s Media (cIMDM) (all from Sigma), 10% FBS, 2mM L-glutamine, 0.5 mM sodium pyruvate, 100 U penicillin, 100 μg streptomycin, and 50 μM 2-ME (all from Gibco, Lifetechnologies). Cells were fixed in 2% paraformaldehyde (Sigma), permeabilized using Permeabilization buffer (Perm, eBioscience) and incubated for 40 minutes with anti-IFN-γ-FITC (XMG1.2), IL-10-PE (JES5-16E3), T-bet-efluor 660 (eBio4B10, eBioscience), or Bcl6-Alexa Fluor 488 (K112-91).

For IL-21 staining, cells were incubated for 40 minutes with recombinant mouse IL-21R-Fc chimera (1 μg, R&D systems, Minneapolis, MN) in Perm and washed twice, followed by 30 min with Alexa Fluor 647 F(ab')₂ goat anti-human IgG (0.3 μg, Fcγ Specific, Jackson ImmunoResearch, West Grove, PA) in Perm buffer. After three washes in FACS buffer, cells were collected on a LSRII Fortessa in the UTMB Flow Cytometry and Cell Sorting Core Facility using FACSDiva software (BDbiosciences, San Jose, CA) and analyzed in FlowJo version 9.7 (TreeStar, Ashland, OR). Compensation was performed in FlowJo using single stained splenocytes (using CD4 in all colors). In adoptive transfer figures, data from 3–4 mice is concatenated to achieve sufficient cell numbers for presentation and Boolean gating analysis, after each mouse was analyzed and averages and SEM calculated. Nuclear co-localization of T-bet and Bcl6 was collected using ImageStream MARKII and analyzed with IDEAS^®^ ImageStream (EMD Millipore, Seattle). For Boolean gating analysis, the distribution of used markers was analyzed with SPICE 5.35 software [28].

### Cell Sorting and Microbead Purification

Splenic CD4^+^ T cells from uninfected mice were enriched using EasySep biotin Selection Kit (STEMCELL, Vancouver, Canada) and a cocktail of biotinylated anti-CD8α (55–6.7), B220 (RA3-6B2), CD11b (MI/70), CD11c (N418), F4/80 (BM8) and Ter119 (all from eBioscience). Enriched T cells were then Naïve (CD44^lo^CD25^-^) sorted with anti-CD4-FITC, CD44-Allophycocyanin-Cy7, and CD25-PE (all eBioscience) on a FACSAriaI with FACDiva software (BDbiosciences). Thy1.1^+^CD4^+^ T cells were isolated via direct magnetic bead separation (Miltenyi Biotec, San Diego, CA) after CD4^+^ T cell enrichment. Cells were washed and resuspended in calcium- and magnesium-free PBS at 10^7^ cells/mL before incubation with 5 μM Cell Trace Violet (CTV, Invitrogen) for 10 minutes at 37°C with shaking, then quenched with FCS. After washing, 2x10^6^ cells were transferred into each mouse i.p.

### Real Time PCR

RNA from Thy1.1^+^CD4^+^ T cells was extracted (RNeasy, Qiagen, Valencia, CA) and treated with DNAse (DNAse I, Invitrogen), adjusted to 40–100 ng/μL and reverse transcribed in a final volume of 20 μL (High capacity reverse transcription kit (Applied Biosystems, Grand Island, NY). Between 2–40 ng of reverse transcribed RNA was amplified using iTaq Universal SYBR Green Supermix (Bio-Rad, Hercules, CA) with the following primer pairs: *bcl6*, forward, 5′ CCGGCACGCTAGTGATGTT 3′, reverse, 5′ TGTCTTATGGGCTCTAAACTGCT 3′, and qSTAR qPCR primer pairs (Origene, Rockville, MD) against *tbx21* (MP216689), *prdm1* (MP211365), *eomes* (MP204243). The level of expression was determined by the comparative threshold method (2^-^ΔΔ^CT^) compared to naïve (CD44^lo^CD25^-^) CD4 T cells from uninfected BAC-In mice as a calibrator sample and the 18s ribosomal RNA (*rp18s*) gene as a reference gene for normalization. Samples were collected using a ViiA7 Real-Time PCR system (Applied Biosystems).

### Statistics

Statistical analysis was performed in Prism (GraphPad, La Jolla, CA) using Student’s *t*-test one-tail or two-tail when indicated. *p*<0.05 was accepted as a statistically significant difference.

## Results

### Kinetics of Th1 differentiation in response to *P*. *chabaudi* infection

**Fig 1:**
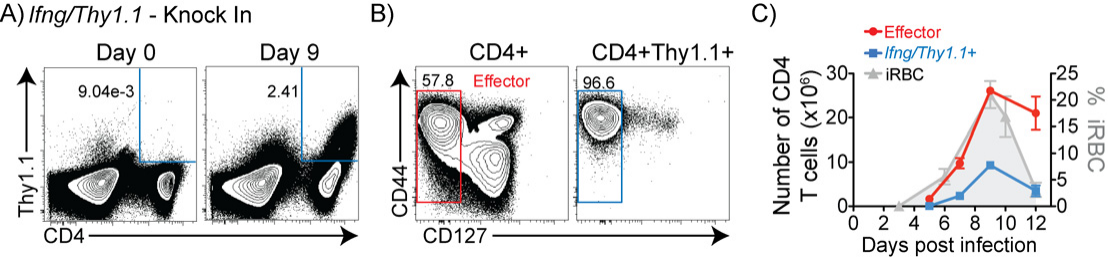
IFN-γ-producing effector cells expand and contract with kinetics similar to parasitemia. *Ifng/Thy1*.*1* Knock-In (KI) mice were infected with *P*. *chabaudi* infected RBC (iRBC). Splenocytes were harvested and analyzed by flow cytometry on various days post-infection (p.i.) and (A) were gated on CD4^+^ *Ifng*/*Thy1*.*1* ^+^, shown at day 0 and 9 p.i. Average percentages from three animals are shown on plots. (B) CD4^+^ *Ifng*/*Thy1*.*1* ^+^ (as gated in A) showing CD4^+^CD127^-^ effector gate (red box) and Thy1.1+CD127- effector gate (blue box) at day 9 p.i. (C) Total numbers of CD4^+^ effector T cells and CD4^+^ *Ifng*/*Thy1*.*1* ^+^ T cells (as gated in B) per spleen overlaid with parasitemia curve (%infected RBCs/total RBCs). Data are representative of eight independent experiments with three animals per timepoint. Error bars represent SEM.

*P*. *chabaudi* malaria infection reaches maximal parasitemia by day 9 and persists at low levels for up to three months [29]. To investigate Th1 differentiation during *P*. *chabaudi* infection, we used IFN-γ reporter (*Ifng/Thy1*.*1* knock-in, KI) mice, where the Thy1.1 reporter gene is expressed transiently during active transcription of *Ifng* [30]. Direct *ex vivo* analysis of splenocytes by flow cytometry on day 9 post-infection (p.i.) revealed a small *Ifng/Thy1*.*1* ^+^ CD4^+^ T cell population (average of 2.41%, **Fig 1A**). The majority of these Thy1.1^+^CD4^+^ T cells had an IL-7Rα negative (96.6% CD127^-^), effector phenotype (**Fig 1B**). Similar expansion kinetics were observed for all Teff and *Ifng/Thy1*.*1* ^*+*^ effector T cells along with parasite levels, which all peaked at day 9 p.i. (**Fig 1C**).

**Fig 2:**
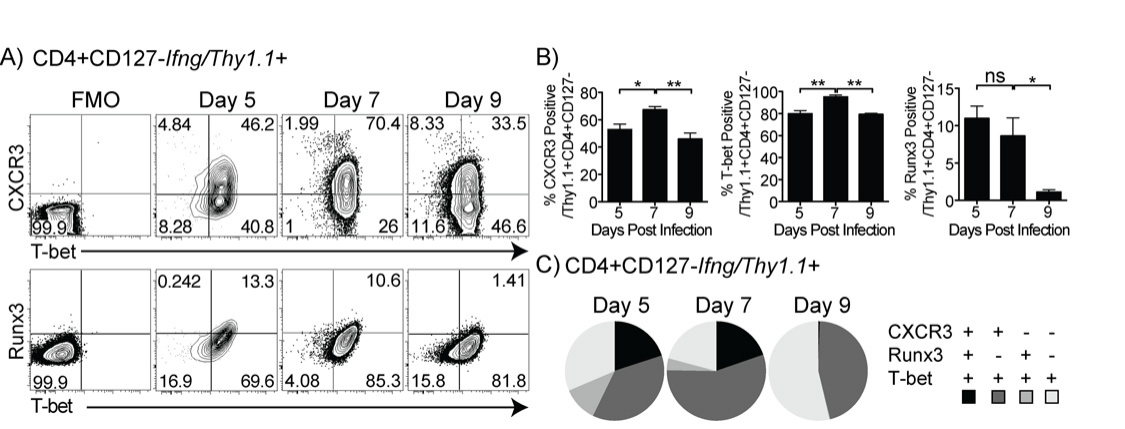
Expression of markers of Th1 differentiation is reduced at the peak of IFN-γ production. *Ifng/Thy1*.*1* KI mice were infected with *P*. *chabaudi* iRBC, and splenocytes were analyzed. (A) Contour plots show expression of CXCR3, T-bet, and RUNX3 gated on CD4^+^ *Ifng*/*Thy1*.*1* ^+^ on days 5, 7, and 9 post-infection. The numbers depicted on the plots are average percentages. Gates were drawn using fluorescence minus one (FMO, CXCR3) or isotype (T-bet, Runx3) controls for each day, as shown to the left. (B) Bar graphs showing percentages of CXCR3, T-bet, and RUNX3 positive cells in Thy1.1^+^ Teff population on each day. Data is summarized in (C) pie charts of Boolean gating analysis of all possible combinations of CXCR3^+^, Runx3^+^, and T-bet^+^ within CD4^+^ *Ifng*/*Thy1*.*1* ^+^ effector T cells. *Ifng/Thy1*.*1* ^+^ T cells expressing all three Th1 markers are shown in black. Two markers are shown in dark grey, and one marker is indicated by light grey. Data are representative of three independent experiments with three animals per timepoint. Statistical significance was obtained using Students *t* test. Error bar represents SEM; ^★^ *p* < 0.05, ^★★^ *p* < 0.01, *ns* = not significant.

In order to investigate the degree of Th1 differentiation of IFN-γ^+^ cells during the effector phase of the response, we measured expression of Th1 markers on CD4 T cells at timepoints leading up to the peak of *P*. *chabaudi* infection. We used T-bet, the master regulator of Th1 differentiation, as a marker of Th1 commitment, since high levels of this transcription factor are expressed in Th1 cells fully committed to making IFN-γ. On day 5 p.i., minimal effector cells were detectable and most of the *Ifng/Thy1*.*1* ^*+*^ cells expressed T-bet (average, 79%) above that of the T-bet isotype control. Further, 55% of the cells were above fluorescence minus one (FMO) multi-color staining control for CXCR3, the IFN-γ-induced homing receptor for inflamed tissues (**Fig 2A**). An average of 47% of Thy1.1^+^CD4^+^T-bet^+^ T cells co-expressed CXCR3 on day 5 p.i. On day 7 p.i., Teff appeared Th1-like, with 95% of Thy1.1^+^CD4^+^ T cells expressing T-bet and an average of 70.4% of Thy1.1^+^ cells co-expressing CXCR3. However, at day 9 p.i., we observed a downregulation of T-bet in *Ifng*/*Thy1*.*1* ^*+*^ CD4 T cells, accompanied by a significant downregulation of CXCR3. However, the downregulation of CXCR3 has been reported to be required for proliferation in CD8 T cells in the red pulp, suggesting this may be independent of IFN-γ production [31]. Runx3, a chromatin-remodeling factor critical for commitment to IFN-γ production by Th1 cells [32], also peaked on day 7, and was downregulated in *Ifng*/*Thy1*.*1* ^*+*^ CD4 T cells by day 9 (**Fig 2A and 2B**). This data is summarized in **Fig 2C** by Boolean gating analysis of the Th1 phenotype of the *Ifng*/*Thy1*.*1* ^*+*^ CD4 T cell population through the peak of *P*. *chabaudi* infection. We found that on day 5 p.i., 16% of the Thy1.1^+^CD4^+^ T cells co-expressed T-bet, CXCR3, and Runx3 with a similar percentage on day 7; on day 9, only 0.5% of the Thy1.1^+^CD4^+^ T cells maintained the expression of these three markers. These results indicate the generation of a strong Th1 response by day 7 p.i., characterized by the expression of T-bet, CXCR3, Runx3, and the production of IFN-γ. However, this is followed by T-bet downregulation, and decreased CXCR3 and Runx3 by day 9 p.i., despite a continued increase in the number of IFN-γ-producing T cells.

### IFN-γ^hi^CXCR5^+^ effector T cells are the main source of IL-21 and IL-10 in *P*. *chabaudi* infection

**Fig 3:**
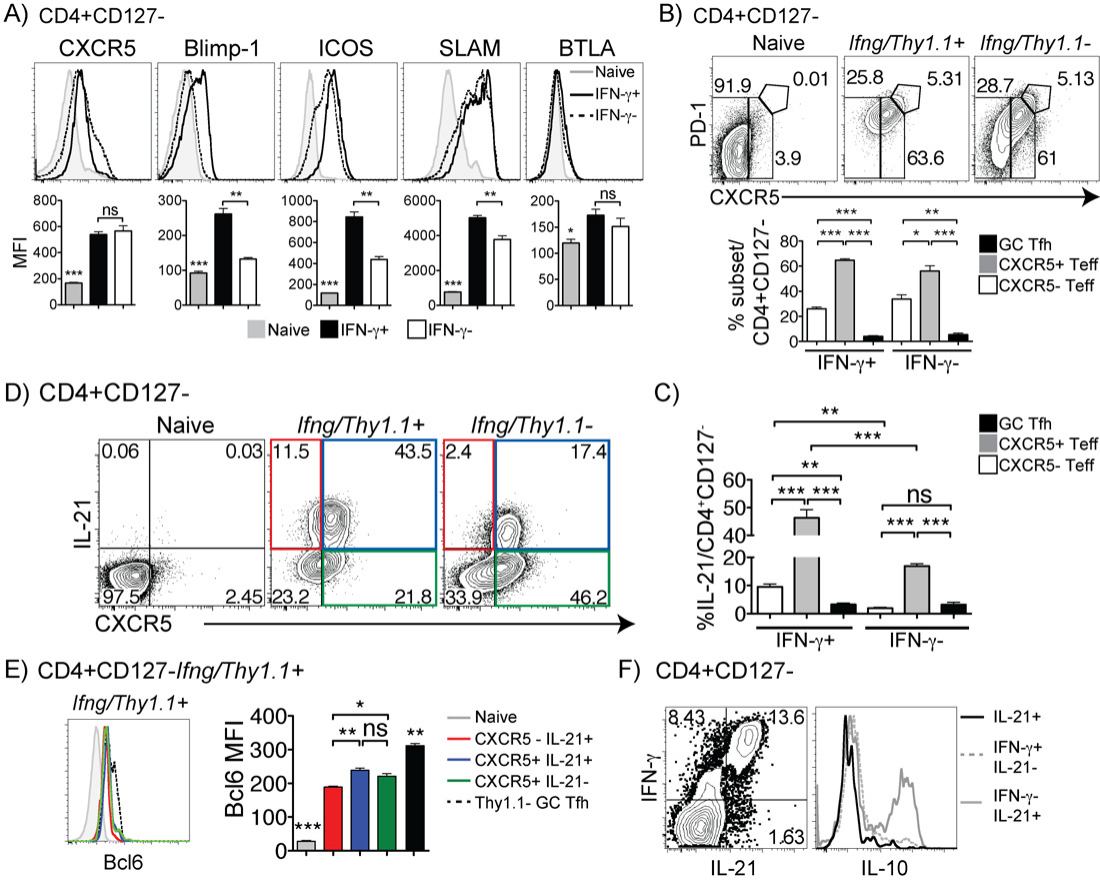
The *Ifng*/*Thy1*.*1* ^+^ T cell population expresses Tfh markers. Splenocytes from day 7 p.i (A) C57Bl/6J mice were analyzed for expression of CXCR5, Blimp-1, ICOS, SLAM, and BTLA in naïve (CD44^lo^CD127^+^, grey), IFN-γ^+^ (black line), and IFN-γ^-^ (dotted line) effector T cells (CD127^-^) as determined by intracellular cytokine staining. Bar graphs show mean fluorescence intensity (MFI). (B) Contour plots of PD-1 and CXCR5 from *Ifng/Thy1*.*1* knock-in (KI) mice effector T cells (CD4^+^CD127^-^, as shown in Fig 2) are gated on naïve T cells from uninfected, *Ifng/Thy1*.*1* ^+^, and *Ifng/Thy1*.*1* ^-^ mice. Bar graph shows percentages of *Ifng/Thy1*.*1* ^+/—^subsets out of total effector T cells. (C) Contour plot of IL-21 and CXCR5 expression. (D) Percentages of IL-21 within effector subsets. (E) Histogram showing Bcl6 expression in CXCR5^-^IL-21^+^ (C, red box), CXCR5^+^IL-21^+^ (C, blue box), and CXCR5^+^IL-21^-^ (C, green box) subsets relative to naïve and *Ifng/Thy1*.*1* ^-^ GC Tfh T cells. Bar graph shows average MFI of Bcl6 staining shown for each subset. (F) Contour plots for IFN-γ, IL-21 and IL-10 by intracellular cytokine staining in C57Bl/6J splenocytes day 7 p.i. Data are representative of four independent experiments with 3 mice per group. Statistical significance was obtained using Students *t* test. Error bar represents SEM; ^★^ *p* < 0.05, ^★★^ *p* < 0.01, ^★★★^ *p* < 0.001, ns = not significant.

Upon investigation of responsive T cells in this infection, we observed a dramatic increase in CXCR5, ICOS, and SLAM. Given the presumed predominance of Th1 cells at this timepoint [4], we investigated expression of several Tfh markers on *Ifng/Thy1*.*1* ^+^ effector T cells. Surprisingly, at day 7 p.i., *Ifng/Thy1*.*1* ^+^ and *Ifng/Thy1*.*1* ^-^ effector T cells expressed comparable levels of CXCR5 and BTLA (B and T Lymphocyte Attenuator), while expression of Blimp-1, ICOS, and SLAM was higher on *Ifng/Thy1*.*1* ^+^ (**Fig 3A**). This unusual profile suggests that these IFN-γ producers also have some features of Tfh cells. To differentiate Tfh and GC Tfh cells, we used CXCR5, the chemokine receptor that determines localization to B cell areas in lymphoid tissue; and PD-1, which is highly expressed on GC-Tfh [33]. Despite a majority of PD-1^int^ Teff, as observed in the CD4 response to chronic *M*. *tuberculosis* [34], we detected both CXCR5^hi^PD-1^hi^ (GC Tfh) cells and CXCR5^+^ (Tfh) cells within the *Ifng/Thy1*.*1* ^+^ effector T cell population on day 7 p.i. (**Fig 3B**). After gating on these populations, we found that IL-21 production by CXCR5^+^ *Ifng/Thy1*.*1* ^+^ effector T cells was greater than in the *Ifng/Thy1*.*1* ^-^ Teff (**Fig 3C and 3D**).

Quantifying the fraction of IL-21 in the three populations, shown in Fig 3B, revealed that CXCR5^+^ IFN-γ^+^ cells generated the most IL-21. These levels were even greater than that produced by CXCR5^hi^PD-1^hi^ GC Tfh cells and CXCR5^-^ effector cells (**Fig 3D**). Moreover, the mean fluorescence intensity (MFI) of IL-21 in *Ifng/Thy1*.*1* ^+^ effector T cells was slightly higher (average, 611) than the *Ifng/Thy1*.*1* ^-^ population (536, p<0.05). Given the similarity of the Th1 cells to Tfh, we measured expression of Bcl6, the Tfh lineage-determining transcription factor, in the *Ifng/Thy1*.*1* ^+^ and IL-21 or CXCR5 expressing populations shown in Fig 3D at day 7 p.i. (**Fig 3E**). All *Ifng/Thy1*.*1* ^+^ effector subsets expressed a higher level of Bcl6 than naïve (CD44^lo^CD127^+^) CD4 T cells, but lower than *Ifng/Thy1*.*1* ^-^ CXCR5^hi^PD-1^hi^ GC Tfh. Intriguingly, we also found that the majority of IL-21 producers also made IFN-γ and IL-10 (**Fig 3F**), and only the IFN-γ^hi^ Th1 cells produced both IL-21 and IL-10.

### Bcl6 T cell deficiency abolishes CXCR5^+^ Germinal Center T follicular helper cells, but not CXCR5^+^IL-21^+^IFN-γ^+^ T cells

**Fig 4:**
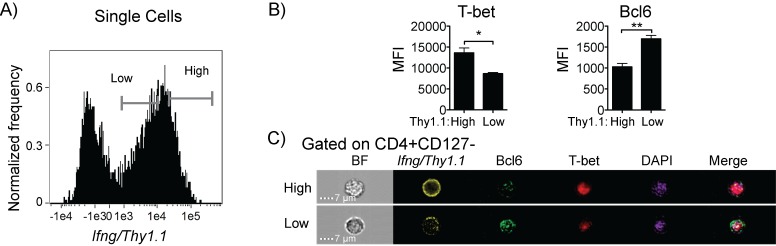
Level of T-bet in the nucleus correlates with *Ifng*/*Thy1*.*1* expression. *Ifng/Thy1*.*1* BAC-In reporter mice were infected with *P*. *chabaudi* and splenocytes were analyzed on day 7 post-infection. After positive selection of Thy1.1 cells, cells were stained for surface CD4, CD127, and Thy1.1 (yellow), followed by intracellular staining for Bcl6 (green) and T-bet (red). Just before analysis, nuclei are stained with DAPI (purple), and analyzed by imaging flow cytometry. (A) Histogram showing *Ifng*/*Thy1*.*1* expression is gated on single focused cells. Cells were gated on CD4^+^CD127^-^ Teff and (B) expression of Bcl6 and T-bet was measured within *Ifng*/*Thy1*.*1* high and low gates as shown in (A). (C) Representative images of individual cells from imaging flow cytometry. Bright Field (BF) and DAPI/Bcl-6/T-bet merged images are shown. Statistical significance was obtained using Students *t* test. Error bar represents SEM; ^★^ *p* < 0.05, ^★★^ *p* < 0.01.

Bcl6 is the primary transcription factor that determines Tfh cell lineage [20]. However, in Th1 cultures, Bcl6 has been shown to be driven by IL-12 and to oppose expression of IFN-γ by direct association with T-bet [35]. Therefore, we examined the co-expression and localization of Bcl6 and T-bet, and tested the correlation of Bcl6 and T-bet expression with *Ifng*, as well as the requirement of Bcl6 to generate T cell populations making IL-21 or expressing CXCR5. To enrich the *Ifng* ^+^ cells for imaging flow cytometry analysis, *Ifng*/*Thy1*.*1* ^+^ cells were purified from infected *Ifng*/*Thy1*.*1* Knock-In reporter animals on day 7 p.i. *Ifng*/*Thy1*.*1* ^*hi*^ and *Ifng*/*Thy1*.*1* ^*lo*^ populations were gated (**Fig 4A**) and expression of Bcl6 and T-bet was quantified (**Fig 4B**). We observed a correlation between high expression of Thy1.1 to high T-bet or low Bcl6. Using the bright detail similarity score, which compares the brightness of two probes in the image, and DAPI to stain the nucleus, we observed both T-bet and Bcl6 in the nucleus of Thy1.1^+^ effector (CD127^-^) T cells (**Fig 4C**). However, in some cells both T-bet and Bcl6 could also be detected in the cytoplasm. This has been previously reported for T-bet [36].

**Fig 5:**
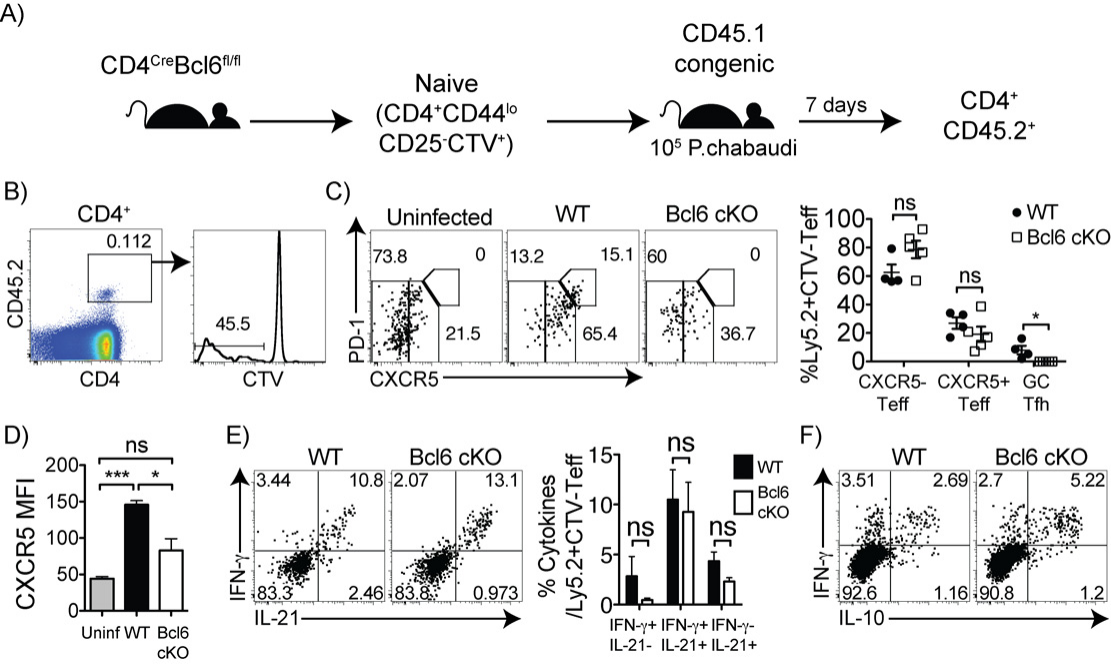
Bcl6 controls generation of GC Tfh, but not cytokine profile, in responding effector cells. (A) Naïve (CD44^lo^CD25^-^) CD4 T cells (2x10^6^) from either Bcl6^fl/fl^ CD4^Cre^ (Bcl6 cKO) or Bcl6^fl/fl^ (WT) were labeled with cell trace violet (CTV) and adoptively transferred into Ly5.1 (CD45.1) congenic mice, followed by *P*. *chabaudi* infection. On day 7 post-infection, splenocytes were harvested and stained with (B) CD4, CD45.2, CTV, (C, D) PD-1, CXCR5, (E) IFN-γ, IL-21, and (F) IFN-γ, IL-10. (B) Plots showing the gating on responding CD4^+^CD45.2^+^CTV^-^ T cells. (C) Graph shows percentages for individual recipients of effector T cell subsets. No CD45.2^+^ CXCR5^hi^PD-1^hi^ GC Tfh cells were detected in any recipient of Bcl6 cKO T cells. (D) Bar graph shows CXCR5 MFI of CD4^+^CD45.2^+^CTV^-^ donor cells. (E) Plots and bar graph of average IFN-γ and IL-21 cytokine producers in the responding donor cells (CD45.2^+^CTV^-^) in recipients of WT and Bcl6 cKO T cells. (F) Dot plot showing intracellular cytokine staining. Data are representative of three independent experiments with 4–5 animals per group. Numbers within plots represent mean percentages. Statistical significance was obtained using Students *t*-test. Error bars represent the SEM; ^★^ *p* < 0.05, ^★★★^ *p* < 0.001, ns = not significant.

As low levels of T-bet coincides with low Bcl6 expression and correlates with reduced *Ifng*/*Thy1*.*1* expression, we tested the role of Bcl6 in induction of the mixed Th1 and Tfh phenotype observed here. We infected Bcl6^fl/fl^Cre^CD4^ conditional KO (cKO), where the Zn finger-encoding exons of the Bcl6 gene are flanked with loxP sites and deleted specifically from all CD4^+^ T cells, as previously verified [25]. This domain of Bcl6 is involved in both DNA binding, and the protein-protein interaction with T-bet [35, 37]. Therefore, the potential Bcl6-mediated regulation of Th1 lineage loci and binding to T-bet are both deficient. As Bcl6 cKO mice do not make Germinal Center B cells [25], which are likely to be essential for clearance of *P*. *chabaudi* [5, 8], we sorted naïve CD4 T cells (CD44^lo^CD25^-^) from Bcl6 cKO mice (CD45.2), labeled them with the CFSE analog cell trace violet (CTV), and adoptively transferred them into CD45.1 congenic mice, followed by *P*. *chabaudi* infection (**Fig 5A**). Both groups of recipients exhibited a similar course of parasitemia (data not shown), and were expected to make antibodies normally. On day 7 p.i., responding CTV^-^CD45.2^+^ effector T cells were identified and the Bcl6- versus wildtype-derived cells were characterized and compared (**Fig 5B**). As expected, Bcl6 deficient T cells did not generate CXCR5^hi^PD-1^hi^ GC Tfh cells (**Fig 5C**). While the frequency of CXCR5^+^ effector T cells did not change between groups, we did observe a decrease in the MFI of CXCR5 on the Bcl6 cKO T cells (**Fig 5D**). However, Bcl6 deficiency had no effect on IFN-γ or IL-21 production (**Fig 5E**). Although we observed a decrease in IFN-γ^**-**^IL-21^+^ cells in this experiment, this effect was not repeatable. There was no change in IFN-γ^+^IL-10^+^ effector T cells in the cKO cells either (**Fig 5F**). Because both groups of recipients are wildtype, cell numbers followed the same trends shown here as percentages. Overall, these data demonstrate that Bcl6 deficiency in T cells reduces GC Tfh formation, as previously described [25]. However, Bcl6 deficiency had no effect on the IFN-γ^+^IL-10^+^IL-21^+^ effector T cells, indicating that these cells are not derived from the Bcl6-dependent Tfh lineage. This was surprising, given that IFN-γ cells express so many markers indicative of Tfh.

**Fig 6:**
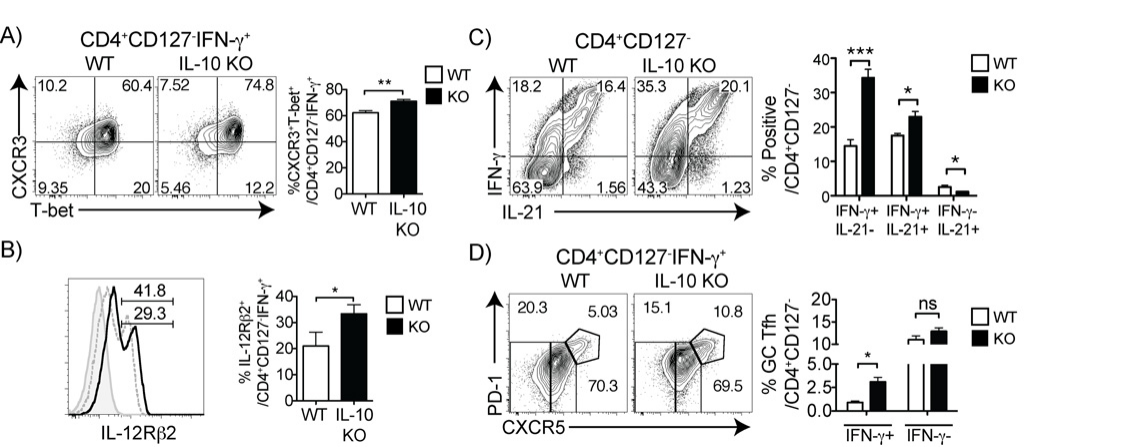
IL-10 regulates generation of both IL-21^+^IFN-γ^+^ and IFN-γ^+^ GC Tfh cells. Wildtype (WT) and IL-10 deficient mice (IL-10 KO) were infected and 7 days later splenocytes were analyzed by intracellular staining. Contour plots and bar graphs from IL-10 KO and WT controls showing expression of (A) CXCR3 and T-bet, and (B) IL-12Rβ2 in the IFN-γ^+^ Teff (CD127^-^) population; (C) IFN-γ and IL-21 in all Teff, and (D) PD-1 and CXCR5 in the IFN-γ^+^ Teff population. Data are representative of two independent experiments with 3–4 animals per group. Statistical significance from Students *t*-test. Error bars represent the SEM; ^★^ *p* < 0.05, ^★★^ *p* < 0.01, ^★★★^ *p* < 0.001), ns = not significant.

In order to determine if these cells are regulated by IL-10, which downregulates IL-12 [38, 39], we infected IL-10 deficient mice. On day 7 of infection, as expected, effector T cells from IL-10 deficient animals showed an increase in the percentage of CXCR3^+^T-bet^+^ Th1 cells within the IFN-γ^+^ effector T cell gate with no difference in cell numbers (**Fig 6A** and data not shown). As IL-12Rβ2 is required for reinforcement of Th1 differentiation [40], we determined that the proportion and number of IL-12Rβ2^+^IFN-γ^+^ effector T cells was significantly increased in the IL-10 deficient mice (**Fig 6B**). Along with this augmented CXCR3^+^T-bet^+^IL-12Rβ2^+^ Th1 phenotype, we observed an increase in the production of IFN-γ single-producers and IFN-γ-IL-21 double-producers, with a significant overall increase in IL-21 production in the IL-10 KO Teff (**Fig 6C**). Interestingly, this phenotype was also accompanied by an increase in cells with the CXCR5^hi^PD-1^hi^ GC Tfh phenotype within the IFN-γ-producing effector T cells, but not within the IFN-γ^-^ subset (**Fig 6D**). As infection and splenomegaly is similar in IL-10 deficient and wildtype animals, cell numbers follow the same trends shown. These results suggest that IL-10 regulates generation of both IL-21^+^IFN-γ^+^ and IFN-γ^+^ GC Tfh cells. Collectively, these data suggest that IL-21, known to be essential for generation of a protective B cell response in *P*. *chabaudi*, is highly produced by Th1 cells.

### T-bet^+^ IFN-γ^+^ T cells decay in the effector to memory transition, but are promoted by chronic infection

**Fig 7:**
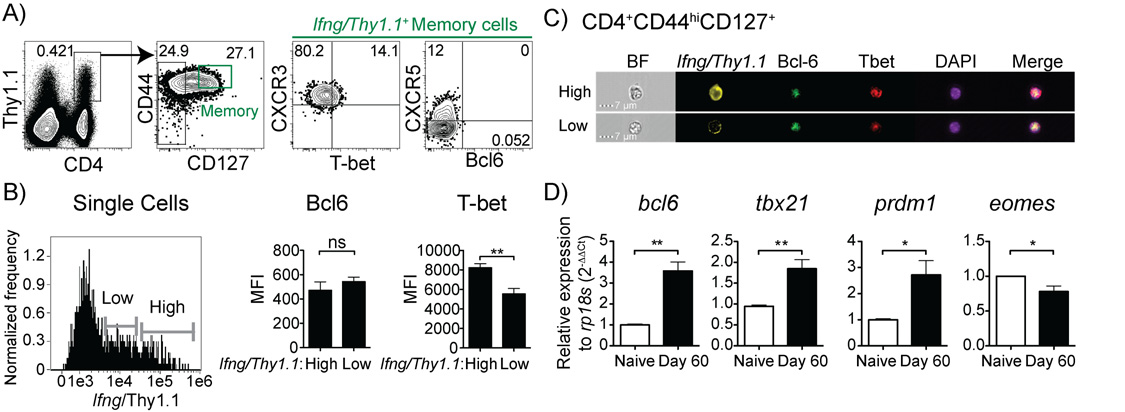
IFN-γ^+^ CD4 memory T cells maintain CXCR5 expression, but little T-bet. BAC-In mice were infected and splenocytes were analyzed by flow cytometry on day 60 post-infection. (A) CD4^+^ *Ifng*/*Thy1*.*1* ^+^ memory (CD44^hi^CD127^+^, green box, gate set on CD4^+^) T cells were gated and expression of CXCR3, T-bet, CXCR5, and Bcl6 was measured. Numbers represent mean percentages. (B, C) Surface staining of CD4^+^ T cells with CD4, CD44, CD127, and Thy1.1 (yellow) was followed by intracellular staining with Bcl-6 (green), T-bet (red), and nuclei with DAPI (purple). Cells were analyzed by imaging flow cytometry. (B) Histogram showing expression of *Ifng*/*Thy1*.*1* on single focused cells. Bar graphs show MFI of Bcl6 and T-bet within Thy1.1 gates (C) Representative images of individual *Ifng*/*Thy1*.*1* high and low cells showing Bcl6 and T-bet expression and localization in relation to the DAPI stained nucleus. Bright field (BF; left) and DAPI (nucleus)/Bcl6/T-bet merged images also shown (right). (D) Real time PCR analysis of *bcl6*, *tbx21*, *prdm1*, and *eomes* of CD4^+^Thy1.1^+^ sorted T cells. Results were normalized to control gene *rp18s*. RNA from FACS Sorted naïve (CD44^lo^CD25^-^) cells from uninfected, aged-matched BAC-In mice was used as control. Data are representative of three (A) and one (B, C, D) independent experiments with 3–4 animals per group. Statistical significance shown using Students *t*-test. Error bars represent the SEM; ^★^ *p* < 0.05, ^★★^ *p* < 0.01, ns = not significant.

We have previously shown that the chronic phase of infection with *P*. *chabaudi* generates memory-phenotype specific CD4 T cells, and maintains excellent protection from re-infection. We have proposed that this improved immunity during chronic infection is mediated by TNF^+^IFN-γ^+^IL-2^-^ Th1 cells, as production of these cytokines depends on the chronic infection [29, 41]. Furthermore, both protection and IFN-γ production decay in *P*. *chabaudi* [4, 42, 43]. Although Th1 memory in this infection is not fully understood, it appears unlikely to be fully committed to make IFN-γ upon restimulation, especially without continuous stimulation. Therefore, we investigated markers of Th1 commitment on *Ifng* ^+^ cells later in infection using a different reporter system. In *Ifng*/*Thy1*.*1* BAC-In mice, cells with an accessible *Ifng* locus can be identified by prolonged expression of Thy1.1 because of an SV40 intron/polyA tail downstream of the *Thy1*.*1* insert that stabilizes mRNA expression [30]. We infected *Ifng*/*Thy1*.*1* BAC-In mice with *P*. *chabaudi*, harvested splenocytes on day 60 p.i., and analyzed expression of Th1 markers in *Ifng*/*Thy1*.*1* ^+^ CD4 T cells. We identified *Ifng* ^+^ memory T cells as CD4^+^ *Ifng*/*Thy1*.*1* ^+^ CD44^hi^CD127^+^ on day 60 p.i., and observed that they all expressed CXCR3, but only 14% expressed T-bet (**Fig 7A**) above the isotype control baseline (**S1 Fig**). The transcription factors Runx3 and Eomesodermin (Eomes) were also undetectable by flow cytometry in the memory Th1 cells (data not shown). Interestingly, the Tfh chemokine receptor CXCR5 was expressed on a significant subset of the *Ifng*/*Thy1*.*1* ^+^ memory T cells (average of 12%), though Bcl6 was not detectable (**Fig 7A**). However, *Ifng* ^+^ CXCR5^+^ and T-bet^+^ populations did not overlap significantly. As imaging flow cytometry is more sensitive than conventional flow cytometry, we analyzed Thy1.1^+^ T cells harvested from BAC-In mice 60 days p.i. by imaging flow cytometry to detect Bcl6 and T-bet (**Fig 7B**). Similar to effector T cells, *Ifng*/*Thy1*.*1* ^*hi*^ memory cells displayed higher levels of T-bet than *Ifng*/*Thy1*.*1* ^*lo*^ cells. On the contrary, while expression of Bcl6 was detectable, it did not change in *Ifng*/*Thy1*.*1* ^*hi*^ cells. Representative images of Thy1.1^hi^ and Thy1.1^lo^ cells showing Bcl6 and T-bet expression are shown in **Fig 7C**. Real-time PCR confirmed expression of both Bcl6 and T-bet (*tbx21*) in the memory population (**Fig 7D**). Notably, we also detected *prdm1* (Blimp-1) mRNA, suggesting that Bcl6 fails to completely repress its target genes at the memory stage. Interestingly, CD8 effector memory cells also express Blimp-1 [44]. *Eomes* mRNA was slightly decreased in the day 60 cells compared to naïve controls.

**Fig 8:**
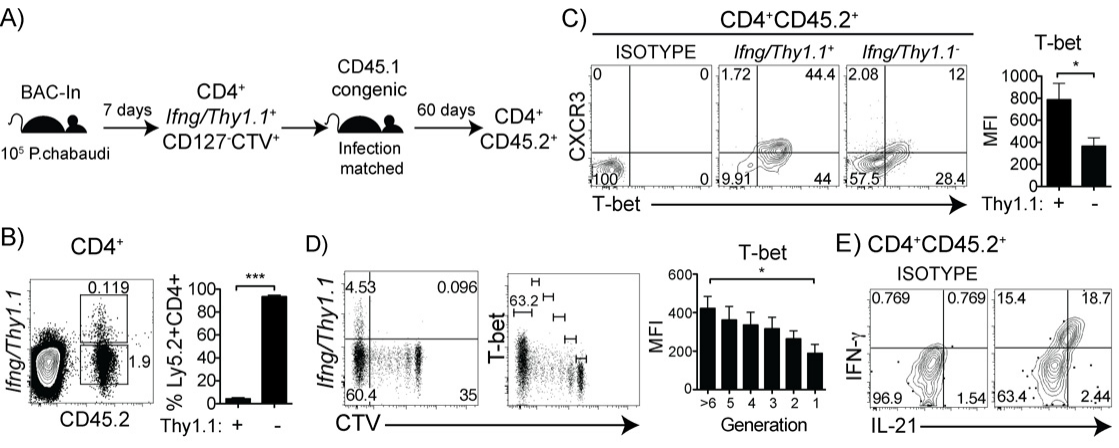
IFN-γ^+^ memory cells co-produce IL-21, and *Ifng*-accessibility and T-bet are maintained by division during chronic malaria infection. (A) BAC-In mice (CD45.2) were infected and at day 7 post-infection, CD4^+^ *Ifng*/*Thy1*.*1* ^+^ T cells were purified, CTV labeled, and adoptively transferred into infection-matched CD45.1 recipients. At day 60 post-infection, splenocytes were analyzed by flow cytometry. (B) Dot plot and bar graph shows expression of *Ifng/Thy1*.*1* in recovered donor cells. (C) Contour plots gated on CD4^+^CD45.2^+^ show expression of CXCR3 and T-bet in recovered *Ifng*/*Thy1*.*1* ^+^ and *Ifng*/*Thy1*.*1* ^-^ populations. Bar graph shows T-bet MFI. (D) *Ifng*/*Thy1*.*1* and T-bet within the CTV^-^ maximally divided cells. Bar graph shows T-bet MFI in each division (E) IFN-γ and IL-21 expression *ex vivo* in CD4^+^CD45.2^+^ donor cells. Data are representative of two independent experiments with 3–4 mice per experiment. Statistical significance shown using Students *t*-test. Error bars represent SEM; ^★^ *p* < 0.05, ^★★★^ *p* < 0.001.

In order to investigate whether the cumulative T-bet^lo^ memory cells observed in the previous experiment are derived from IFN-γ^+^ effector T cells, we adoptively transferred *Ifng*/*Thy1*.*1* ^+^ effector T cells (99.7% purity) from *Ifng*/*Thy1*.*1* BAC-In animals on day 7 p.i. into infection-matched CD45.1 recipients, as illustrated in **Fig 8A**. At day 60 p.i., we collected recipient splenocytes and analyzed the CD45.2^+^ (formerly Thy1.1^+^) memory cell phenotype by flow cytometry. Surprisingly, we observed that the majority of the cells that were transferred from day 7 infected donors, had downregulated Thy1.1 by day 60 p.i (>94%, **Fig 8B**). The loss of *Ifng*/*Thy1*.*1* expression suggests a less accessible *Ifng* locus, and was accompanied by a significant decrease in CXCR3 and T-bet expression in *Ifng*/*Thy1*.*1* ^*-*^ *cells* (**Fig 8C**). However, the *Ifng*/*Thy1*.*1* ^+^ T cells maintained both CXCR3 and T-bet expression, suggesting a stronger Th1 phenotype in this small fraction of the recovered cells. When cells transferred at day 7 were labeled with CTV, we observed that by day 60 p.i. an average of 78% of the transferred cells had divided more than six times. Essentially only these cells included *Ifng/Thy1*.*1* ^+^ cells and contained the highest levels of T-bet (**Fig 8D**). Taken together, these findings suggest that Th1 commitment, as defined by T-bet expression, is maintained by division in this infection. Interestingly, after *ex vivo* restimulation we found that a significant fraction of effector cells that survived into the memory phase still co-produced IFN-γ and IL-21 at day 60 p.i. (**Fig 8E**). These data suggest that the mixed Th1/Tfh population entered the memory pool, and that maintenance of IFN-γ production and T-bet expression by Th1 cells is linked to further parasite-driven proliferation.

## Discussion

The mechanisms of concomitant immunity, which is defined as protection from reinfection during persistent infection, are poorly understood. In acute parasitic infection, fully committed Th1 cells can be generated [45, 46]. However, in chronic parasitic infection, T cells in the memory phase require IL-12 for continuous IFN-γ production, which suggests that a lack of intrinsic commitment to IFN-γ production is promoted by chronic antigen stimulation [47–49]. Here, in a model of malaria infection that lasts for up to 90 days [29], we studied commitment to IFN-γ production by effector CD4 T cells and their phenotype in the memory phase. The present data demonstrate that during the effector response to *P*. *chabaudi* infection, responding IFN-γ-producing T cells do not maintain a robust Th1 (T-bet^+^ CXCR3^+^ Runx3^+^) phenotype. Instead, Teff in this infection consist largely of IFN-γ^+^ Tfh-like cells that only maintain expression of IFN-γ and T-bet into the memory phase with proliferation. Weak maintenance of the Th1 phenotype into the memory phase was evident as only 6% of adoptively transferred IFN-γ^+^ Teff maintained *Ifng*-accessibility, T-bet, and CXCR3 expression. Importantly, Bcl6, IL-21, and CXCR5 expression persisted in the IFN-γ^+^ cells as well. Our data showing a loss of T-bet expression in the absence of proliferation supports evidence that IFN-γ^+^ T cells decay over time after exposure to malaria, correlating with the documented loss of immunity with time observed in animals [42]. This is also reported in humans with reduced parasite-exposure upon emigration from endemic areas [50]. Therefore, maintenance of IFN-γ production by Th1 memory cells in malaria depends on antigen or cytokines generated by chronic infection, as in other parasitic infections [43, 47–49]. This data highlights the challenge of classifying these protective Th1 cells as effector, long-lived effector, or long-lived effector memory T cells, despite some evidence supporting each of these conclusions [41, 51–53]. Studies of T cell immunity in chronic viral infection suggest that the landscape of the T cell response to chronic infection includes IFN-γ^+^ IL-21^+^ multifunctional cytokine producers and T helper cell phenotype plasticity [8, 54–58]. Fahey *et al*. have shown that prolonged TCR stimulation during a persistent viral infection can re-direct Th1 cells towards the Tfh lineage in a TCR-dependent manner [59], however, they did not investigate expression of IL-21 in this context.

Here, we confirm that IL-10 and IL-21 are both produced by IFN-γ^+^ CXCR5^+^ T cells [8]. This suggests that the IFN-γ^+^IL-10^+^ double-producing Teff population previously shown to protect animals from pathology in *P*. *chabaudi* and *P*. *yoelli*, are the same population that also enhance the B cell response via IL-21 [7, 8]. IL-21 has been shown to be critical for isotype-switched antibody production and parasite clearance in this infection [8]. We also show that in addition to CXCR5, IFN-γ^+^IL-10^+^IL-21^+^ T cells express the Tfh markers Bcl6, ICOS, BTLA, and SLAM. However, we have now shown that generation of the majority of IL-21 producers is Bcl6-independent, suggesting that they are not of the Tfh lineage. The IFN-γ^+^IL-21^+^ cells are also increased in an IL-10 deficient environment, which promotes Th1 development. Interestingly, IFN-γ^+^ GC Tfh were increased in this context as well. This data suggests that the majority of the Teff in *P*. *chabaudi* infection are Th1-type cells that express many of the markers of Tfh, and may be similar to those defined in chronic LCMV as “exhausted” due to reduced homeostatic proliferation [54], which is also a feature of effector memory T cells (Tem) [60]. Maintenance of Tem has also been shown to depend on ICOS [61]. Previous studies demonstrated a phenotypic overlap between Th1 and Tfh cells during *Toxoplasma gondii* infection, where T-bet expression was required for downregulation of IL-21 and CXCR5 to achieve Th1 commitment [62].

We also observed a separate, very small population of IFN-γ^+^ cells that expressed high levels of CXCR5 and PD-1, generated IL-21, and were regulated by Bcl6, thus, confirming their GC Tfh lineage. The B cell response to *P*. *chabaudi* begins with a strong extrafollicular antibody producing cell response and IgM, followed by a delayed specific IgG response. While IL-21 and CXCR5, widely considered Tfh-related molecules, are both predominantly expressed by IFN-γ^+^ cells in this infection, this population is not regulated by Bcl6. Importantly, both types of multifunctional T cell populations (IFN-γ^+^CXCR5^+^ and IFN-γ^+^IL-21^+^) entered the memory pool. For this reason, the memory cell population likely maintains T helper function through the expression of IL-21 and CXCR5. Some surviving cells showed improved maintenance of *Ifng-*locus accessibility, and expressed T-bet, but this correlated with extensive division after the effector phase. Importantly, the majority of memory T cells that maintained IFN-γ expression produced higher levels of IL-21 *ex vivo*, as well.

While there is a predominantly Th1 cytokine profile in CD4 T cells in healthy rural African individuals who are exposed frequently to malaria [63], these T cells also express high levels of IL-10 and IL-21. Interestingly, both cytokines are co-expressed with IFN-γ, though the three together were not tested in the human studies. In children with acute *P*. *falciparum* infection, increased plasma IL-21 levels correlated with IgG1 and IgG3 antibodies and the development of clinical immunity [64]. In *Plasmodium spp*., IL-10 is induced in Th1 cells by IL-27 [7, 65]. Moreover, a previous study has shown that IL-21 can also be induced through this pathway [16], suggesting a mechanism for the generation of these multi-cytokine producers. In other types of chronic diseases, IFN-γ-producing cells are also the major source of IL-21 [24, 66, 67], although, in those studies the cells were also CXCR5^+^PD-1^hi^. In a study of chronic LCMV, CD4 T cells had a weak Th1 transcriptional profile compared to those from acute infection. Moreover, none of the other T helper lineages were favored in these T cells, suggesting increased plasticity in the face of chronic infection [54]. This chronically stimulated population also made IFN-γ and TNF-α, as well as IL-10 and IL-21. Importantly, in chronic LCMV infection, IL-21 is required to prevent CD8 exhaustion sustaining effector activity [55, 56, 58], however, its effect on chronic CD4 responses is less clear [7, 68, 69].

Bcl6 is considered the lineage-defining transcription factor of the Tfh subset [22]. Bcl6 regulates Th subset differentiation by inhibiting Th1, Th2, Th17, and Treg transcription factors and cytokine genes [70]. However, Tfh cells can acquire cytokine profiles and maintain the master-regulatory transcription factors of other Th subsets, such as IFN-γ, IL-4, or IL-17, suggesting flexibility in this less committed subset [23]. This data suggests a new paradigm where Tfh cells co-express Bcl6 and also another lineage-defining transcription factor so that the function of CD4 T cells avoid terminal differentiation and remain responsive to chronic infection producing cytokines. While CXCR5^hi^PD1^hi^ GC Tfh cells, which can make IL-21, differentiate in a Bcl6-dependent manner [25], Tfh markers are not solely controlled by Bcl6. For example, IL-21 production by Tfh cells is controlled by STAT3 and c-Maf [71], and c-Maf can also induce CXCR5 expression [19]. Furthermore, IFN-γ production by CD4 T cells from Bcl6 cKO mice in response to sheep red blood cells was not affected [25]. It is interesting to note that *in vitro* Th1 differentiation induces IL-21 production without expression of CXCR5 [18]. In agreement with this data, we found that only the IFN-γ^+^CXCR5^hi^PD-1^hi^ GC Tfh population was regulated by Bcl6. Therefore, the CXCR5^+^ Tfh-like phenotype found in IFN-γ^+^ cells is likely to be a consequence of Bcl6-inducing cytokines (such as IL-6, IL-21 or IL-27) or B:T cell interactions involving ICOS, which is also highly upregulated on all effector cells in this infection, and is reported to promote Tem survival [61].

The majority of IL-21 was produced by the IFN-γ^+^CXCR5^+^ T cell subset in accordance with Peréz-Mazliah [8]. Infection of IL-10 KO animals results in increased Th1 polarization [39], however, the proportion of the CXCR5^hi^PD-1^hi^ GC Tfh subset within the IFN-γ^+^ population also increased significantly in these animals. This suggests that IL-10 also regulates the CXCR5^hi^PD-1^hi^ IFN-γ^+^ GC Tfh phenotype. Additionally, there is precedence for IL-10 regulating CXCR5^hi^BTLA^hi^ Tfh cells [72].

In summary, memory T cells in this infection show poor maintenance of *Ifng/Thy1*.*1* expression in the BAC-In reporter animals. This was accompanied by low T-bet expression, and continuous expression of IL-21 and Bcl6. This conclusion was presaged by previous work in this infection that indicated poor Th1 commitment, as measured by IL-4 production and help for malaria-specific antibody by T cells late in infection [4]. Interestingly, T-bet is not essential for IFN-γ production in *Plasmodium yoelii* infection, and actually inhibits parasite killing [73, 74]. Therefore, we propose that the mixed Th1/Tfh phenotype reported here may actually be a beneficial response. Indeed, plasticity in T cells has been shown to benefit the host in tuberculosis infection [75], and this mechanism is still being investigated. Our data is relevant for the understanding of protective responses required for development of effective protective vaccines to control malaria pathology.

## Supporting Information

S1 Fig FMO and isotype controls for staining in Ifng/Thy1.1 BAC-In. BAC-In mice were infected and splenocytes were analyzed by flow cytometry on day 60 post-infection. CD4^+^ *Ifng*/*Thy1*.*1* ^+^ memory (CD44^hi^CD127^+^, gate set on CD4^+^) T cells were gated and isotype controls for T-bet, and Bcl6 and FMO for CXCR3, CXCR5 is shown.
(TIF)

## Abbreviations

Tfh: follicular Th cell
Teff: effector T cell
PD-1: programmed death-1
Tmem: memory T cell
KO: knockout
cKO: conditional knockout
GC: germinal center
p.i.: post-infection
i.p.: intraperitoneal
iRBC: infected Red Blood Cell
BF: Bright Field
FMO: Fluorescence Minus One
MFI: Mean Fluorescence Intensity
Tem: effector memory T cell
