# Correction: IFN-γ and IL-21 Double Producing T Cells Are Bcl6-Independent and Survive into the Memory Phase in *Plasmodium chabaudi* Infection

**DOI:** 10.1371/journal.pone.0155570

**Published:** 2016-08-22

**Authors:** Victor H. Carpio, Michael M. Opata, Marelle E. Montañez, Pinaki P. Banerjee, Alexander L. Dent, Robin Stephens

There is an error in the ninth sentence of the second paragraph of the “Bcl6 T cell deficiency abolishes CXCR5+ Germinal Center T follicular helper cells, but not CXCR5+IL-21+IFN-γ+ T cells” section of the Results. The correct sentence is: However, only a fraction of the CXCR5+ Teff are Bcl6-dependent, though we observed a decrease in the MFI of CXCR5 on the Bcl6 cKO T cells (Fig 5D).

There is an error in the third sentence of the third paragraph of the Discussion section. The correct sentence is: While IL-21 and CXCR5, widely considered Tfh-related molecules, are both predominantly expressed by IFN-γ+ cells in this infection, this population is only slightly affected by deficiency of Bcl6.

There is an error in the ninth sentence of the fifth paragraph of the Discussion section. The correct sentence is: In agreement with this data, we found that primarily the IFN-γ+CXCR5+PD-1^hi^ GC Tfh population was regulated by Bcl6.

There is an error in the second sentence of the Acknowledgements section. The correct sentence is: We are grateful for the kind advice of Jean Langhorne and Ken Murphy, and the provision of Ifng /Thy1.1 animals from Casey Weaver and Laurie Harrington.
