# Correction: IFN-γ and IL-21 Double Producing T Cells Are Bcl6-Independent and Survive into the Memory Phase in *Plasmodium chabaudi* Infection

**DOI:** 10.1371/journal.pone.0174048

**Published:** 2017-03-10

**Authors:** Victor H. Carpio, Michael M. Opata, Marelle E. Montañez, Pinaki P. Banerjee, Alexander L. Dent, Robin Stephens

In Fig 5, the graphs of Fig 5C lack percentages for individual recipients of effector T cell subsets. Please see the updated Fig 5 here.

**Fig 5:**
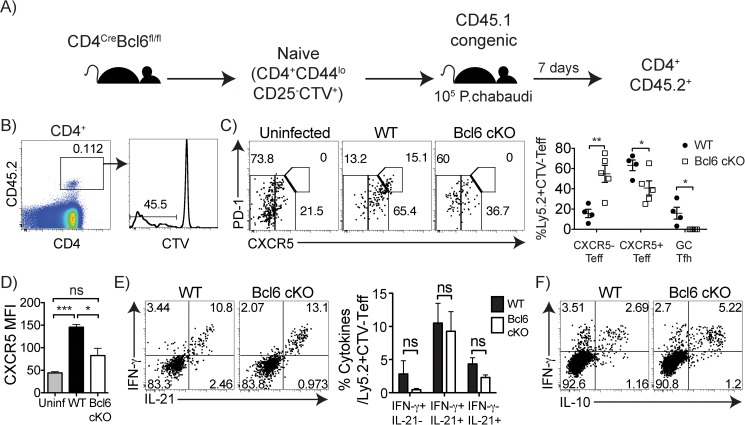
Bcl6 controls generation of GC Tfh, but not cytokine profile, in responding effector cells. (A) Naïve (CD44^lo^CD25^-^) CD4 T cells (2x10^6^) from either Bcl6^fl/fl^ CD4^Cre^ (Bcl6 cKO) or Bcl6^fl/fl^ (WT) were labeled with cell trace violet (CTV) and adoptively transferred into Ly5.1 (CD45.1) congenic mice, followed by *P*. *chabaudi* infection. On day 7 post-infection, splenocytes were harvested and stained with (B) CD4, CD45.2, CTV, (C, D) PD-1, CXCR5, (E) IFN-γ, IL-21, and (F) IFN-γ, IL-10. (B) Plots showing the gating on responding CD4^+^ CD45.2^+^ CTV^-^ T cells. (C) Graph shows percentages for individual recipients of effector T cell subsets. No CD45.2^+^ CXCR5^hi^PD-1^hi^ GC Tfh cells were detected in any recipient of Bcl6 cKO T cells. (D) Bar graph shows CXCR5 MFI of CD4^+^ CD45.2^+^ CTV^-^ donor cells. (E) Plots and bar graph of average IFN-γ and IL-21 cytokine producers in the responding donor cells (CD45.2^+^ CTV^-^) in recipients of WT and Bcl6 cKO T cells. (F) Dot plot showing intracellular cytokine staining. Data are representative of three independent experiments with 4–5 animals per group. Numbers within plots represent mean percentages. Statistical significance was obtained using Students *t*-test. Error bars represent the SEM; *p < 0.05, ***p < 0.001, ns = not significant.
