# Supplementary figures and images for: IFN-γ and IL-21 Double Producing T Cells Are Bcl6-Independent and Survive into the Memory Phase in Plasmodium chabaudi Infection
Source: PLoS One. 2015 Dec 8;10(12):e0144654. doi: 10.1371/journal.pone.0144654 (PMC4672895; doi:10.1371/journal.pone.0144654)

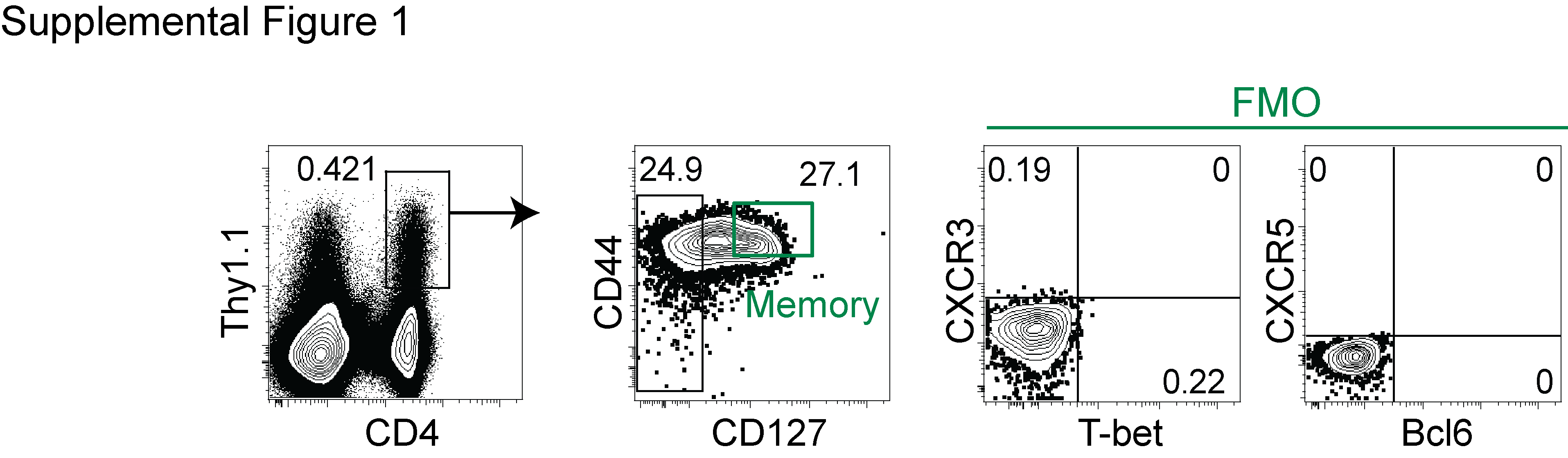

Supplement: S1 Fig — BAC-In mice were infected and splenocytes were analyzed by flow cytometry on day 60 post-infection. CD4+ Ifng/Thy1.1 + memory (CD44hiCD127+, gate set on CD4+) T cells were gated and isotype controls for T-bet, and Bcl6 and FMO for CXCR3, CXCR5 is shown. (TIF) [file pone.0144654.s001.tif]
